# Individual and community factors contributing to anemia among women in rural Baja California, Mexico

**DOI:** 10.1371/journal.pone.0188590

**Published:** 2017-11-27

**Authors:** Molly A. Moor, Miguel A. Fraga, Richard S. Garfein, Hooman H. Rashidi, John Alcaraz, Donna Kritz-Silverstein, John P. Elder, Stephanie K. Brodine

**Affiliations:** 1 Division of Epidemiology and Biostatistics, Graduate School of Public Health, San Diego State University, San Diego, CA, United States of America; 2 Division of Epidemiology, Department of Family Medicine and Public Health, School of Medicine, University of California San Diego, La Jolla, CA, United States of America; 3 School of Medicine, Universidad Autónoma de Baja California, Tijuana, Mexico; 4 Division of Global Public Health, Department of Medicine, School of Medicine, University of California San Diego, La Jolla, CA, United States of America; 5 Department of Pathology and Laboratory Medicine, School of Medicine, University of California Davis, Sacramento, CA, United States of America; 6 Division of Health Promotion and Behavioral Science, Graduate School of Public Health, San Diego State University, San Diego, CA, United States of America; Universidade de Sao Paulo, BRAZIL

## Abstract

**Introduction:**

Anemia is a public health concern among women in rural Baja California, Mexico. The purpose of this study was to identify the individual and community factors contributing to the disproportionately high prevalence of anemia among women in this region.

**Methods:**

A cross-sectional study of 118 women (15–49 years) was performed in a rural *colonia* (small settlement) in Baja California, Mexico in 2012. Participants completed a survey comprised of demographic, socioeconomic, health, and dietary questions and provided a capillary blood sample. A portable HemoCue was used to measure hemoglobin and diagnose anemia. Anemic participants provided a venous blood sample for laboratory testing to elucidate the etiology of anemia. Anemic participants received vitamin supplements and nutritional counseling. Assessments of six local *tiendas* (community grocery stores) were performed to ascertain the types of food available for purchase within the community.

**Results:**

Prevalence of anemia was 22% among women; laboratory tests revealed iron deficiency was the primary etiology in 80.8% of anemia cases. Other causes of anemia in women included vitamin B-12 deficiency (11.5%) and combined iron and vitamin B-12 deficiency (7.7%). Women from low SES households and women enrolled in the government assistance program *Prospera* were significantly more likely to be anemic (OR = 3.48, 95% CI 1.35–8.98 and OR = 2.49, 95% CI 1.02–6.09, respectively). Vitamin supplementation was significantly more common among non-anemic women (OR = 0.12, 95% CI 0.02–0.94). Dietary assessments showed limited consumption of iron absorption enhancing foods such as fruits and vegetables. Assessments of local *tiendas* revealed at least one type of meat and citrus fruit available for purchase at each store; however, leafy green vegetables were only available for purchase at one store.

**Conclusion:**

All cases of anemia were due to nutritional deficiencies. While vitamin supplementation is a temporary solution, improved individual nutrition knowledge and community access to iron absorption enhancing foods, particularly produce, is needed. Promoting government assistance programs like *Prospera* and implementing additional programs designed to improve nutrition and health literacy, in conjunction with ensuring access to nutritious foods, might reduce the high prevalence nutritional anemia within the community.

## Introduction

Anemia is a significant public health problem in developing countries, and occurs more frequently in pre-school age children and women of childbearing age [1,2]. National studies show the prevalence of anemia in Mexico has decreased in the past decade [3–6] and is currently 11.6% among non-pregnant women, 17.9% among pregnant women [4,5]. Evidence indicates that anemia prevalence has decreased as a result of the implementation of government programs [6]. Of particular relevance is a national Mexican program, *Prospera* (formerly called *Oportunidades*), which provides food subsidies, cash incentives, and healthcare services to impoverished families in designated communities to improve quality of life [7,8]. Enrollment in *Prospera* has been associated with decreased anemia prevalence [3,8,9], with one study indicating that children who received assistance from *Prospera* were 25.5% less likely to be anemic than children who did not receive benefits from the program [10].

While programs have been implemented to improve the health of Mexican citizens, anemia rates still remain higher among Mexicans residing in rural areas as compared to those living in urban environments [11]. In a prior study conducted by our institutions, we examined anemia prevalence among women living in a rural, farming *colonia* (small settlement) in Baja California, Mexico. Results revealed an anemia prevalence rate of 23.3% among women, which is substantially higher than the national average [12]. However, the study provided only limited information concerning the etiology of anemia. Evidence from dietary recall and blood smears collected in the previous study showed that iron deficiency was the probable cause; however, other contributing factors could not be dismissed. While iron deficiency is the most common cause of anemia [2,13], studies show that nutrient deficiencies including vitamin B-12 and folate [14,15], as well as lead toxicity due to use of lead-glazed painted cookware [16], may contribute to anemia among Mexicans. The purpose of this study was to determine the etiology of anemia, and to identify the individual and community factors contributing to a high prevalence of anemia among women in rural, Baja California, Mexico.

## Materials and methods

A cross-sectional study was conducted during a two-day period in October 2012 in a rural *colonia* located near San Quintín, Baja California, Mexico. This study was performed in conjunction with VIIDAI (Viajes Interinstitucional de Integración, Docente, Asistencia y de Investigación/ Inter-institutional Field Experiences for Integration, Teaching, Medical Service, and Research), a binational public health training program between San Diego State University (SDSU), University of California San Diego (UCSD), and Universidad Autónoma de Baja California, Tijuana (UABC) [12,17,18,19]. VIIDAI affords public health and medical students an opportunity to provide supervised clinical and public health services to underserved communities. Twice per year, VIIDAI provides a free, 2-day clinic where local community members receive free healthcare services; this study was conducted in conjunction with a VIIDAI clinic. Approval for this study was granted by the SDSU, UCSD, and UABC Institutional Review Boards. Written informed consent was obtained from all study participants, and written parental consent was obtained for all study participants who were under the age of 18 years.

### Study setting

San Quintín is located in an agricultural region of Baja California, approximately 200 miles south of the California, USA/Baja California, Mexico border. Several small, rural, *colonias* are located near San Quintín that are populated primarily by indigenous peoples who migrated from southern Mexico to work in the agricultural fields [20] and have since established permanent residency in the region due to the consistent availability of work. As of the 2010 census, approximately 3800 individuals reside in the *colonia* where this study was conducted [21]. The *colonia* where the study was conducted has been the site of ongoing VIIDAI clinic and public health interventions for the past decade [12, 17–19].

### Sampling techniques

The study population included women of childbearing age (15–49 years). Participants were recruited in one of two settings: the free, 2-day VIIDAI clinic or the participant’s home. In the clinic setting, random sampling was performed to screen women for study participation while waiting in line to see a provider. For household enrollment, a map of the *colonia* was used to randomly select blocks of homes in the *colonia* for inclusion in the study. Each household in the designated block was approached for study participation, beginning at the northeast corner and continuing in a clockwise manner until all houses on the block were approached. Individuals were eligible for the study if they were women between the ages of 15–49 years with the ability to speak Spanish. One woman per household was allowed to participate in the study. If more than one woman was eligible, the eligible individuals rolled a pair of dice, and the woman who rolled the highest number was included in the study. Persons were not eligible if their primary residence was in a different community or they did not meet the age criteria. We enrolled as many participants as possible during the 2-day data collection timeframe.

### Survey instrument

An English questionnaire containing demographic, socioeconomic, and health factors putatively related to anemia was developed specifically for this study, translated into Spanish, and then back translated into English to confirm accuracy. The Spanish survey was then pilot tested among Spanish-speaking indigenous persons to ensure comprehension. Administration of the survey among study participations occurred via face-to-face interviews at the clinic or in the participants’ homes. Ownership of specific household items including a refrigerator, vehicle, radio, television, and phone were used to create a socioeconomic status (SES) score, with one point given for ownership of each item. Thus, SES scores ranged from 0–5; a score of 0–2 was considered low SES. Dietary intake data was based on a yes or no response to a 48-hour recall of the consumption of specific food items and beverages. Occupation was categorized in three ways: ‘agricultural’ referred to a person employed as a field laborer, a ‘homemaker’ was a female not currently working outside of the home, and ‘other’ included business owners or women employed outside of the home, but not working in the agricultural industry. Literacy was defined by an individual’s self-reported ability to read a newspaper and write a letter. Additional questions concerning attendance at the VIIDAI medical clinics and participation in government assistance programs were also asked. All indigenous women who were approached for study and surveyed were fluent in Spanish; thus all surveys were conducted in Spanish.

### Collection of blood samples & diagnosis of anemia

Anemia testing was performed among all participants. Each study participant provided a capillary blood sample obtained by finger prick. The initial diagnosis of anemia was performed by measuring hemoglobin (Hb) with a portable HemoCue photometer (Hemocue AB, Angelholm, Sweden). Non-pregnant women 15–49 years of age with Hb levels of less than 12.0 g/dl, and pregnant women with Hb levels of less than 11.0 g/dl were considered anemic [22]. A sample of venous blood was collected from women diagnosed with anemia, and blood smears were also prepared. The blood was collected in vacutainer tubes and kept refrigerated until delivery to a laboratory where complete blood count (CBC) with differential, serum iron, iron saturation, total iron binding capacity (TIBC), transferrin, unsaturated iron binding capacity (UIBC), ferritin, vitamin B-12, folate, and lead tests were performed. A hematopathologist reviewed blood smears and laboratory results to classify anemia and determine etiology. *Mild anemia* was defined as Hb levels of 11.0–11.9 g/dl for non-pregnant women, and 10.0–10.9 g/dl for pregnant women. *Moderate anemia* was defined as 8.0–11.0 g/dl for non-pregnant women, and 7.0–10.0 g/dl for pregnant women. *Severe anemia* was defined as Hb levels below 8.0 g/dl for non-pregnant women, and below 7.0 g/dl for pregnant women [22]. Blood lead levels were tested in all anemic individuals and values of equal to or greater than 10 ug/dL were used to diagnose lead poisioning [23]. Isolated iron deficiency anemia was diagnosed by low serum iron (below 35 ug/dL), low transferrin saturation (below 15%), low ferritin (below 13 ng/mL), high TIBC (above 450 ug/dL), or high UIBC (above 375 ug/dL). Anemia due to vitamin B-12 deficiency was defined as having vitamin B-12 levels below 211 pg/mL. Folate deficiency was defined as folate levels below 3.1 ng/mL [24]. In cases where some laboratory parameters may have appeared within normal limits, participants were evaluated for chronic diseases and/or early iron deficiency anemia.

Individuals diagnosed with anemia received a personalized consultation from a dietician associated with the VIIDAI clinic. The dietician educated participants regarding the causes of anemia and shared dietary modifications to increase nutrient consumption and absorption as a mechanism for preventing anemia.

### Grocery store assessments

Observational *tienda* (small, local grocery store) assessments served as a mechanism to examine the quantity and quality of food available for consumption by community residents. No supermarkets are located within the *colonia*, and the closest supermarket is over 5 miles away; thus, many residents, especially those without a vehicle, utilize *tiendas* as their primary source to purchase food. There are approximately 15 *tiendas* situated within the *colonia*. To ascertain the types of foods available for purchase, 6 (40%) of these *tiendas* were randomly selected and visited. All *tienda* owners or employees approached for inclusion in the study granted verbal permission for a visual inspection of their store. Data collected included store size, capacity, and food items available for purchase. The size of the store was classified by the number of aisles and registers; small stores had 2 aisles or less, medium stores had 3–4 aisles, and large stores had at least 5 aisles. Stores were also considered large in size if they contained more than one cash register. Approval to survey each store was obtained from the individual who was responsible for overseeing the store on the day of the visit. A total of 6 *tiendas* were approached for study inclusion and all agreed to participate.

### Statistical analysis

Continuous variables were checked for normality and descriptive statistics were calculated for all variables. Chi-square tests were used to examine categorical variables and independent sample t-tests were used to examine continuous variables to assess the differences in anemic and non-anemic individuals. Unadjusted odds ratios (OR) with 95% confidence intervals (CI) were computed to determine associations with anemia status.

A separate stratified analysis was performed to determine if enrollment in *Prospera* was an effect modifier for dietary intake and anemia status. We elected to perform this evaluation because participation in *Prospera* increases a household’s income; thus, enrolled households may have additional money to purchase food or vitamin supplements.

A multivariate logistic regression analysis was also performed and included variables that were significantly associated with anemia status in the bivariate analysis. All statistical tests were two-tailed; p-values less than 0.05 were considered statistically significant. Data were analyzed using PASW Statistics version 18.0 (SPSS Inc., Chicago, IL). A post-hoc power analyses was also calculated using G*Power version 3.1.6 (Franz Faul, Kiel University, Kiel, Germany).

## Results

### Participant characteristics

A total of 118 women (9 (7.6%) of whom were pregnant) completed the anemia survey and underwent anemia testing; 40 women (35%) were enrolled from the clinic, the remaining individuals were enrolled from community households. The mean age of women in the study was 29.7±9.0 years. Results showed that the overall anemia prevalence was 22% among both pregnant and non-pregnant women (Table 1). There was no significant difference in prevalence of anemia by location of enrollment (Table 2). Among those women who were anemic, the majority of cases were mild or moderate in severity (Table 1).

**Table 1:** Prevalence and severity of anemia in women living in a rural farming community in Baja California, Mexico [2012].

	Non-Pregnant Women	Pregnant Women
	(n = 109)	(n = 9)
	n (%)	n (%)
Anemic^a^	24 (22.0)	2 (22.2)
Severity^b^		
Mild	7 (29.2)	1 (50.0)
Moderate	15 (62.5)	1 (50.0)
Severe	2 (8.3)	0 (0)

^a^Anemia was classified as having Hb of <12.0 g/dL among non-pregnant women and <11.0 g/dL among pregnant women.

^b^Anemia severity among non-pregnant women (15–49 yrs) was classified as follows: Mild Anemia = 11.0–11.9 g/dL, Moderate Anemia = 8.0–10.9 g/dL, Severe Anemia = < 8.0 g/dL. For pregnant women (15–49 yrs), the following anemia classifications were used: Mild Anemia = 10.0–10.9 g/dL, Moderate Anemia = 7.0–9.9 g/dL, Severe Anemia = < 7.0 g/dL

**Table 2:** Associations between demographic, SES, and health variables and anemia status in women living in a rural farming community in Baja California, Mexico [2012] (n = 118).

Variable	Anemic (n = 26) mean±SD or n (%)	Not Anemic (n = 92) mean±SD or n (%)	OR (95% CI)	p
*Demographic Characteristics*				
Age	30.3 ± 9.0	29.5 ± 9.1	1.01 (0.96–1.06)	0.681
Survey Location^a^				0.403
Home	18 (72.0)	56 (62.9)		
Clinic	7 (28.0)	33 (37.1)	0.66 (0.25–1.75)-	
Occupation				0.141
Agriculture^b^	10 (38.5)	34 (37.0)	-	
Homemaker	16 (61.5)	46 (50.0)	1.18 (0.48–2.92)	0.717
Other	0 (0)	12 (13.0)	-	
Low SES	11 (42.3)	16 (17.4)	3.48 (1.35–8.98)	0.010
Speak Indigenous Language	7 (26.9)	27 (29.3)	0.89 (0.33–2.5)	0.810
Education Completed				0.811
None^b^	4 (15.4)	12 (13.0)	-	
Primary	16 (61.5)	53 (57.6)	0.91 (0.26–3.20)	0.878
Secondary or more	6 (23.1)	27 (29.3)	0.67 (0.16–2.80)	0.580
Literacy	23 (88.5)	81 (88.0)	1.04 (0.27–4.05)	0.954
Uses painted cookware^a^	2 (8.0)	6 (6.7)	1.22 (0.23–6.44)	0.817
*Maternal & Health Characteristics*				
Number of Pregnancies	2.6 ± 1.7	3.0 ± 2.3	0.91 (0.73–1.13)	0.385
Currently Pregnant	2 (7.7)	7 (7.6)	1.01 (0.20–5.19)	0.989
Gave Birth ≤6 months	2 (7.7)	5 (5.4)	1.45 (0.27–7.95)	0.669
Currently Breastfeeding^a^	3 (13.0)	15 (16.9)	0.74 (0.20–2.81)	0.658
Enrolled in *Prospera*	16 (61.5)	36 (39.1)	2.49 (1.02–6.09)	*0*.*046*
Visited VIIDAI clinic ≤5yrs	19 (73.1)	59 (64.1)	1.52 (0.58–3.99)	0.397
Received nutrition education	17 (65.4)	62 (67.4)	0.94 (0.37–2.29)	0.848
*Dietary Characteristics*				
Foods Consumed ≤48 hrs				
Fish	8 (30.8)	33 (35.9)	0.80 (0.31–2.03)	0.630
Beef	19 (73.1)	77 (83.7)	0.53 (0.19–1.48)	0.224
White Meat	22 (84.6)	78 (84.8)	0.99 (0.30–3.30)	0.983
Eggs	25 (96.2)	83 (90.2)	2.71 (0.33–22.45)	0.355
Cereal	19 (73.1)	63 (68.5)	1.25 (0.47–3.30)	0.653
Nuts	6 (23.1)	21 (22.8)	1.01 (0.36–2.85)	0.979
Legumes	26 (100)	91 (98.9)	-	-
Dried Fruit	7 (26.9)	21 (22.8)	1.25 (0.46–3.37)	0.665
Grains, tortillas, pasta	25 (96.2)	91 (98.9)	0.28 (0.02–4.55)	0.367
Green, leafy vegetables	10 (38.5)	49 (53.3)	0.55 (0.23–1.34)	0.186
Beverage Intake ≤48 hrs				
Tea	12 (46.2)	40 (43.5)	1.11 (0.47–2.67)	0.808
Coffee	19 (73.1)	66 (71.1)	1.07 (0.40–2.84)	0.893
Citrus Juice	14 (53.8)	67 (72.8)	0.44 (0.18–1.07)	0.069
Alcohol	1 (3.8)	2 (2.2)	1.80 (0.16–20.67)	0.637
Vitamin Supplement Use	1 (3.8)	23 (25.0)	0.12 (0.02–0.94)	0.043

^a^Data were missing for some variables and the total sample size for each variable is as follows: currently breastfeeding (n = 112), survey location (n = 114), use of painted cookware (n = 115)

^b^Indicates reference category

### Demographic characteristics

Demographic and household characteristics were compared between anemic and non-anemic women. Anemic women were more likely (OR = 3.48, 95% CI 1.35–8.98) to reside in a low SES household than women who were not anemic (Table 2). Of note, education level was uniformly low among women, with greater than two-thirds receiving no more than an elementary education. No differences were found between clinic and household participants with respect to demographic or SES characteristics (data not shown). Other comparisons between anemic and non-anemic participants are shown in Table 2.

### Maternal characteristics & participation in health programs

With respect to participation in health programs, anemic women were more likely (OR = 2.49, 95% CI 1.02–6.09) to be enrolled in the government program *Prospera*, as compared to non-anemic women (Table 2). Attendance at a VIIDAI clinic within the past 5 years was 66.1% among all women regardless of anemia status. However, no differences in VIIDAI clinic attendance rate were found between anemic and non-anemic women (Table 2). Overall, 66.9% of females reported receiving nutrition education either from VIIDAI programs intended to decrease anemia or from outside sources such as *Prospera*, but nutrition education was not associated with decreased likelihood of being anemic (Table 2).

## Dietary characteristics

Dietary intake was compared between anemic and non-anemic women. Significantly lower rates of anemia were observed among women reporting vitamin use (OR = 0.12, 95% CI 0.02–0.94, Table 2). Vitamin use was 66.7% among pregnant women and 16.5% among non-pregnant women (p = 0.002). Of note, vitamin use was not associated with participation in *Prospera* (p = 0.512) or visiting a VIIDAI clinic (p = 0.583). The consumption of citrus juice (containing vitamin C), which enhances the body’s ability to absorb iron, approached significance as being protective against anemia (OR = 0.44, 95% CI 0.18–1.07, Table 2). While no statistically significant differences in food and drink intake were found among anemic and non-anemic women, consumption of citrus juice (a source of vitamin C) was somewhat higher among non-anemic women (Table 2). There was no significant difference in food intake based on enrollment in *Prospera* (data not shown).

Qualitative feedback from the dietician who provided personalized nutrition counseling to anemic women revealed that many women were unfamiliar with anemia; thus, they were also subsequently unaware of strategies for improving iron consumption and absorption via diet. At the conclusion of the counseling session, participants demonstrated improved knowledge of iron-rich foods and iron-enhancing foods. The participants also verbalized that they found the session helpful and planned to prepare nutritious meals that would improve their iron intake and absorption.

### Multivariate results

Logistic regression simultaneously examining the associations of SES, enrollment in *Prospera*, and vitamin supplement use showed that after adjustment, each variable remained independently and significantly associated with anemia. Women from low SES households were more likely (OR = 4.37, 95% CI 1.51–12.60) to be anemic compared to women from higher SES households. Odds of being anemic were 3.59 times higher (95% CI 1.32–9.78) among women enrolled in *Prospera* compared to women not enrolled in *Prospera*. The odds of having anemia were lower (OR = 0.11, 95% CI 0.01–0.90) among women who used vitamin supplements compared to those who did not use vitamins (Table 3). No significant interactions were found between the variables.

**Table 3:** Multivariate logistic regression analysis of demographic, SES, and health variables associated with anemia in women living in a rural farming community of Baja California, Mexico [2012] (n = 118).

Variable	Odds Ratio	95% CI	p
Low SES	4.37	1.51–12.60	0.006
Enrolled in *Prospera*	3.59	1.32–9.78	0.012
Vitamin Supplement Use	0.11	0.01–0.90	0.039

### Laboratory findings

Twenty-six women were anemic and laboratory blood tests showed 18 cases of microcytic anemia, four cases of macrocytic anemia, and four cases of normocytic anemia. Seventeen of the 18 cases of microcytic anemia showed clear evidence of iron deficiency anemia, and the remaining case exhibited both iron deficiency and vitamin B-12 deficiency. No peripheral blood smear or laboratory evidence of concomitant thalassemia was noted in the iron deficient individuals. Three of four cases of macrocytic anemia had vitamin B-12 deficiency, and one case had a vitamin B-12 deficiency and iron deficiency. In all four of the normocytic anemia cases, ferritin was on the lower end of the reference range and TIBC was on the higher end of reference range, which is consistent with early iron deficiency anemia. Among women, iron deficiency accounted for 65.4% of anemia cases, vitamin B-12 deficiency was implicated in 11.5% of anemic women, 7.7% of females had both iron and vitamin B-12 deficiencies, and 15.4% of anemia cases were attributed to early iron deficiency anemia (Table 4). Etiology of anemia among the two pregnant women was iron deficiency and vitamin B-12 deficiency. No cases of elevated blood lead levels were found among women.

**Table 4:** Etiology of anemia among women in a rural farming community of northern Baja California, Mexico [2012] (n = 26).

	Women (15–49 yrs) n (%)
Iron deficiency	17 (65.4)
Early Iron deficiency	4 (15.4)
B-12 deficiency	3 (11.5)
Iron + B-12 deficiency	2 (7.7)
Lead	0 (0)

## Grocery store assessments

Assessments were performed in six *tiendas* (1 small, 2 medium, and 3 large). Five *tiendas* had one cash register, and one *tienda* had two cash registers. General characteristics of the capacity and departments of the *tiendas* are as follows: all stores had refrigerator doors (range 1–9 doors), four stores had freezer doors (three stores had one freezer door, one store had two), all stores sold produce, and three stores sold raw meat (only 2 of these had a butcher service counter). Dry food items were available for purchase at all *tiendas* and included fortified dried cereal, legumes, and enriched pasta. Perishable goods including milk and eggs were available for purchase at all stores; however, meat selection (sources of iron and vitamin B-12) was limited with four stores selling chicken, three stores offering beef and turkey, and two stores selling pork. No fresh fish, frozen fish, or organ meats were found in any of the stores, but canned sardines and tuna were available for purchase at all stores. While iron absorption enhancing produce containing vitamin C was available at all stores, the selection was limited. At least one variety of citrus fruit was offered at each store; however, leafy green vegetables were only available for purchase at one store. A wide assortment of pre-packaged, high caloric, low nutrient ‘junk food’ items were also available for purchase at all stores.

## Discussion

With anemia prevalence nearly double the national average among non-pregnant women [4,5], anemia is a public health concern in this *colonia*. Low SES and enrollment in *Prospera* were significantly associated with anemia among women, whereas vitamin supplement use was protective against anemia. These findings suggest that poverty, in conjunction with limited nutritious food available for purchase at local *tiendas*, contribute to the high prevalence of nutritional anemia in this community. While the presence of *Prospera* in the *colonia* implies that government support is available to improve the health and SES of qualifying households, its effectiveness is unclear. Our findings suggest that perhaps a community effort to improve the penetration of *Prospera*, offering individualized nutritional education and promoting health literacy, as well as collectively working together as a community to enhance access to nutritious foods and encouraging the individual selection of nutritious foods, may be mechanisms by which future interventions could be tailored to decrease the prevalence of anemia in this *colonia*.

Iron deficiency was the primary cause of anemia among women in our study. This is consistent with other studies delineating iron deficiency as the primary etiology of anemia in Mexico [15,25]. Because nutritional deficiencies were the primary cause of anemia in this *colonia*, it is not surprising anemia rates were significantly lower among women who reported taking vitamin supplements. This finding is in accord with a previous study reported that 18% of Mexican women use vitamin supplements and that these supplements have been shown to be protective against anemia [26].

A previous study of in this *colonia* revealed that women in the community who consumed four or more servings of leafy green vegetables per week (a good source of vitamin C which enhances the body’s ability to absorb iron) were less likely to be anemic than women who consumed three or fewer servings of leafy green vegetables per week [12]. While poor diet appears to be a contributing factor to anemia within this community, no significant associations were evident between food items consumed within the past 48 hours and anemia status in the present study. This could be due to the fact that serving sizes and amounts were not assessed, as well as the fact that the study was underpowered to detect dietary differences. A study measuring dietary intake among women from central Mexico showed that iron deficiency was not necessarily due to low iron intake, but instead attributed to lower intake of foods containing non-heme iron (vegetables, grains, and nuts) and foods containing vitamin C (which enhances the body’s ability to absorb iron) [27]. An intervention conducted in central Mexico which included the twice-daily addition of 25 mg of vitamin C to meals found that iron absorption improved from 6.6% at baseline to 22.9% after 2 weeks [27]. Interestingly, in our study the consumption of citrus juice (a source of vitamin C) was much higher among non-anemic women (72.8%), as compared to anemic women (53.8%). This finding suggests that increasing vitamin C intake, and encouraging the simultaneous consumption of iron and vitamin C, may be a feasible method for improving iron absorption and decreasing iron deficiency anemia.

Contrary to previous studies that found enrollment in *Prospera* was protective against anemia among children [3,8]; our study found that women enrolled in the program were significantly more likely to be anemic. There are several plausible explanations for this finding. First, the duration of enrollment in *Prospera* was not obtained; thus, the females in our study who were also enrolled in *Prospera* may not have received monetary and health benefits for a long enough time period to show tangible improvement in health measures such as anemia. Secondly, we found that the poorest households in the community were not necessarily the households that were enrolled in the *Prospera* program. Long-term program enrollment may have helped the lowest SES households to move higher on the SES spectrum as a result of stipends received from participation. Unfortunately, the amount of money each household received from their enrollment in *Prospera* was also unknown among participants in this study. Public data indicates the stipends range from $10 to over $150 USD per month based on maternal characteristics (i.e. currently pregnant or lactating), as well as the age, gender, and education level of children living in the household [28]. Within this community, the difference in monthly aid received from *Prospera* could have a large impact on a family’s ability to purchase food and other items since the majority of households have a weekly income of less than $150 USD [19].

We were unable to account for the lack of differences in food consumption and anemia status based on *Prospera* enrollment, but speculate that women enrolled in *Prospera* may not be using their stipends to purchase nutritious foods which may result from the limited availability of nutritious food available for purchase. Grocery store surveys revealed limited availability of fresh produce within the community; thus, it is possible that the money provided to households from *Prospera* is not used to purchase nutritious foods. One study found that improvements in nutrition as a result of enrollment in *Prospera* may actually be due to intake of fortified food supplements, as opposed to dietary changes in food consumption within the home [29].

Although 65% of anemic women reported receiving nutrition education, the majority of anemic participants who received personalized nutrition counseling were not familiar with anemia or its contributing dietary factors. Considering that the majority of women in the study completed no more than an elementary education, it is possible that the nutrition education they received during their tenure in the community may not have included anemia or anemia prevention strategies. Based on the positive feedback from the participants who attended nutrition counseling focused on anemia prevention, it may be reasonable to offer opportunities for individuals to increase their nutrition education and health literacy by participating in educational offerings that are presented at an appropriate literacy level.

Despite many community members working in the agricultural industry, most fresh fruits and vegetables are not consumed locally because they are exported to the United States for consumption [30]. Mexico is the second largest supplier of agricultural goods to the US, with fresh vegetables and fruit accounting for the majority of agricultural exports [30]. Since produce commands a higher market price in the US, it is more profitable for them to be exported rather than sold locally within Mexico. Similar to other studies [6,11], we found that women of low SES were more likely to be anemic than women of higher household SES, which may indicate that price, as well as availability, of fresh produce make regular consumption more difficult. Our study showed no difference in dietary intake based on enrollment in *Prospera*, suggesting that the ability to consume nutritious foods may be a result of limited food availability in the community. Even with the additional income provided by *Prospera*, if local *tiendas* do not sell fresh produce, being enrolled in *Prospera* may not improve one’s diet.

Although this study successfully identified factors associated with anemia in a community with a history of a high prevalence of this disease, it has several limitations. The two-day data collection period resulted in a small overall sample size. While the study found significant associations between anemia and SES, *Prospera* enrollment, and vitamin consumption, the precision of these measures was limited by the small sample size. Furthermore, the study was underpowered to detect differences among some variables, particularly dietary characteristics. Post-hoc power analyses showed that 80% power was not achieved for any of the examined associations between the dietary intake variables and anemia status. The cross-sectional design impeded the exploration of causal relationships between the duration of enrollment in *Prospera* and anemia status, as well as the extent to which the *Prospera* stipend improves SES and the consumption of nutritious foods. We also do not know how many of the households in our study met the inclusion criteria for the *Prospera* program, but were not enrolled. Furthermore, the *Prospera* program is primarily designed to benefit children. Our study, however, did not gather information about children, but rather focused on women. Consequently, we could not evaluate the impact of the program on children within the community. Another potential shortcoming of the study is recall bias, specifically for dietary intake. Because participants were asked to report food and beverages consumed in the past 48 hours, recall inaccuracies may have impacted the ability to distinguish associations between food consumption and anemia. Despite this weakness, there were dietary trends supporting the laboratory evidence of anemia due to nutritional deficiencies. Adding C-reactive protein as part of the laboratory measures would have provided additional information. Furthermore, obtaining venous blood samples from all participants, rather than just anemic individuals, would have been beneficial in uncovering the overall prevalence of nutrient deficiencies in the community and revealing if all community members, not just anemic individuals, would benefit from future nutritional interventions.

## Conclusion

In summary, all cases of anemia in this community were attributable to nutritional deficiencies. While vitamin supplementation is a temporary solution, improving individual knowledge and community access to iron absorption enhancing foods, particularly produce, is needed in this community. Offering health literacy and nutrition education interventions at an appropriate literacy level is one method of improving individual knowledge. Encouraging local *tiendas* to label nutrient-rich foods to promote the individual purchase of healthy foods by community members, is another feasible option. Additionally, promoting government assistance programs like *Prospera* in the community and implementing additional programs designed to improve nutrition and health literacy, in conjunction with ensuring access to nutritious foods, might serve as an additional mechanism to decrease the high prevalence nutritional anemia within the community. Evidence illustrates that reducing anemia prevalence rates among community members will require a multifaceted approach involving both individual and community aspects.

## Supporting information

S1 File English survey. This is the English version of the survey administered to women in the community.
(DOCX)

S2 File Spanish survey. This is the Spanish version of the survey administered to women in the community.
(DOCX)
