# Supplementary material for: Individual and community factors contributing to anemia among women in rural Baja California, Mexico
Source: PLoS One. 2017 Nov 27;12(11):e0188590. doi: 10.1371/journal.pone.0188590 (PMC5703514; doi:10.1371/journal.pone.0188590)
Supplement: S1 File — This is the English version of the survey administered to women in the community. (DOCX) [file pone.0188590.s001.docx]

**Anemia Prevalence in a migrant colonia in Baja California, Mexico.**

| Name of interviewer _______________________  Institution: UABC □_1_ SDSU □_2_ UCSD □_3_ | Date: ___________________________  Location: Clinic □_1_ House □_2_ |
| --- | --- |

**READ ALOUD:** Can we talk to an adult female (15-49yrs)?

**NEW OR RETEST**

In the past **2 days**, has anyone from this home had a blood analysis (finger prick) specifically for anemia and answered survey questions either in your home or at the school clinic?

⬜_0_ New - Answered “no”, continue interview

⬜_1_ Retest- Answered “yes”, STOP interview

**Read consent form to participant. Keep the signed form and give participant a blank copy to keep.**

**Randomization Exercise**

|  | 1. How many females between 15-49 yrs live in your house? (including yourself) |
| --- | --- |
| # Living in Household |  |

If there is only 1 eligible participant, proceed to Section A.

--Or--

If there is more than one eligible participant, **READ ALOUD**: *One person can be included in this study. All of the eligible women will roll a dice and whoever rolls the highest number will be included in the study.*

**SECTION A: DEMOGRAPHIC & HOUSEHOLD DATA**

| 2. Initials of persons living in household and participating in the survey (from randomization exercise) | 3. Female  (circle) | 4. Date of Birth  mo/day/yr | 5. Age |
| --- | --- | --- | --- |
|  | Yes No | __ __ / __ __ / __ __ | yrs |

Only one response should be entered for each question unless otherwise specified. CIRCLE responseS codes to each question, unless otherwise specified.

***READ ALOUD:*** *I am going to ask you some questions about your life and where you live***.**

| 6. What do you currently do for work? (If more than one category applies, select which one you do most often) | Agricultural farm worker  Home maker  Education (e.g., teacher)  Business employee  Business owner  Student  None  Other: Specify: _________________  REFUSED | 1  2  6  10  11  12  13  14  99 | 6.  ______ |
| --- | --- | --- | --- |
| 7.What is your marital status? | Single/ never married  Married/common law  Divorced  Separated  Widowed  REFUSED | 0  1  2  3  4  99 | 7.  ______ |
| 8. How many people, including yourself, currently live in your household? | WRITE THE NUMBER OF PEOPLE IN THE BOX TO THE RIGHT |  | 8.  ______ |
| 9. How many people living in your household work outside of your home? | Enter # of people |  | 9.  ______ |
| 10. What languages do you speak, even if not very well? **(**READ OUT LIST, CHECK ALL THAT APPLY**)**  YES: 1 NO: 0 | (a) Spanish  (b) English  (c) Triqui  (d) Mixteca  (e) Other: Specify: _________________  REFUSED | 1 / 0  1 / 0  1 / 0  1 / 0  1 / 0  99 | 10.(a) ______  (b) ______  (c) ______  (d) ______  (e) ______  Yes: 1 No: 0 |
| 11. What language is most commonly spoken in your household? | Spanish  English  Triqui  Mixteca  Other: Specify: _________________  REFUSED | 1  2  3  4  5  99 | 11.  ______ |
| 12. Can you read a letter or newspaper? | No  Yes  REFUSED | 0  1  99 | 12.  ______ |
| 13. What is the highest level of schooling that you have completed? | None  Primary  Secondary  High School  More than High School  REFUSED | 0  1  2  3  4  99 | 13.  ______ |
| 14. How many rooms are in your home? | Enter # of rooms |  | 14.________ |
| 15. Do you or someone in your household own a car or truck? | Yes  No  REFUSED | 1  0  99 | 15.  ______ |
| 16. Does your household have the following: (read all ITEMS & mark EACH ITEM AS HAVE OR DO NOT HAVE)  HAVE: 1  DO NOT HAVE: 0 | (a) Electricity  (b) Radio  (c) Television  (d) Refrigerator  (e) Telephone  REFUSED | 1 / 0  1 / 0  1 / 0  1 / 0  1 / 0  99 | 16.  (a) ______  (b) ______  (c) ______  (d) ______  (e) ______ |
| 17. What type of cookware is used in this household?  1=USED 0=NOT USED | Metal  Ceramic  Painted Glazed Ceramic  Other: Specify____________________  Don’t Know  Refused | 1 / 0  1 / 0  1 / 0  1 / 0  88  99 | 17.(a) ______  (b) ______  (c) ______  (d) ______ |

***READ ALOUD:*** *I am going to ask you some questions about your health****.***

**SECTION B: HEALTH**

| 18. ¿Do you receive money from the government for food and health through the *Prospera* program? | No  Yes | 0  1 | 18.  _________ |
| --- | --- | --- | --- |
| 19. We offer a free medical clinic at the school in Apr and Oct each year. ¿Have you ever visited the temporary medical clinic? | No (skip to question 21)  Yes | 0  1 | 19.  __________ |
| 20. How many times have you visited the temporary medical clinic at the school in the last 5 years? | (Write the number in the box to the right) |  | 20.  __________ |
| 21. Have you ever exchanged your glazed ceramic for new cookware while you have lived in this community? | No  Yes | 0  1 | 21.  ­­­  __________ |
| 22. Have you ever received nutrition education or healthy récipes while you have lived in this community? | No  Yes | 0  1 | 22.  ­­­  __________ |

| 23. How would you rate your health? (read all options and select one OF THE FOLLOWING:)  0=Poor 1=Fair 2=Good 3=Excellent | 23.  ______ |
| --- | --- |
| 24. In the past 3 months, have you had any of the following symptoms? (CIRCLE YES OR NO) | Y=yes N=no |
| a. Unexplained weight loss | y _1_ n _0_ |
| b. Weakness | y _1_ n _0_ |
| c. Fatigue | y _1_ n _0_ |
| d. Shortness of Breath | y _1_ n _0_ |
| e. Headache | y _1_ n _0_ |
| f. Dizziness | y _1_ n _0_ |
| g. Irritability | y _1_ n _0_ |
| h. Pale Skin | y _1_ n _0_ |
| i. Increased Heart Rate | y _1_ n _0_ |
| j. Diarrhea. (3 or more loose, watery, or bloody stools per day.) | y _1_ n _0_ |

**SECTION C: reproductive Health**

| 25. Are you currently pregnant? | Yes  No  Don’t know | 1  0  88 | 25.  ______ |
| --- | --- | --- | --- |
| 26. Do you menstruate? (not post-menopausal or pre-menarche) | Yes  No | 1  0 | 26.  ______ |
| 27. How many times in your life have you been pregnant, whether or not you had the baby? | Enter # of PREGNANCIES  (if none, put ‘0’& skip tO SECTION D) |  | 27.  ______ |
| 28. Have you had any children in the past 6 months? | Yes  No | 1  0 | 28.  ______ |
| 29. Are you currently breastfeeding? | Yes  No (skip to question 47)  NOT APPLICABLE | 1  0  77 | 29.  ______ |

**SECTION D: Food**

| 30. | *For the following questions, please tell me if you ate each food in the past 48 hours.* | **Si_1_** | **No_0_** |
| --- | --- | --- | --- |
| a. | Fish or seafood | □_1_ | □_0_ |
| b. | Beef | □_1_ | □_0_ |
| c. | Turkey, chicken, pork | □_1_ | □_0_ |
| d. | Egg | □_1_ | □_0_ |
| e. | Cereal or instant cereals | □_1_ | □_0_ |
| f. | Nuts or seeds | □_1_ | □_0_ |
| g. | Beans, lentils, and legumes | □_1_ | □_0_ |
| h. | Dried fruit | □_1_ | □_0_ |
| j. | Rice, bread, pasta, tortillas | □_1_ | □_0_ |
| k. | Green Vegetables (such as asparagus, broccoli, peas, spinach) | □_1_ | □_0_ |
| l. | Tea | □_1_ | □_0_ |
| m. | Coffee | □_1_ | □_0_ |
| n. | Orange juice or citrus fruits (oranges, lemons, grapefruit) | □_1_ | □_0_ |
| o. | Alcohol | □_1_ | □_0_ |
| p. | Vitamins | □_1_ | □_0_ |

***READ ALOUD:*** *Thank you, now we will test to see if you are anemic.*

****SECTION E: HEMOCUE RESULTS****

| 31. Hemocue Test Result (enter # from HemoCue) |  |
| --- | --- |
| 32. Was the individual anemic and referred for blood draw? | y _1_ n _0_ |

HEMOCUE TEST:

HemoCue Machine #: _____________ Person who performed test: _________________

WRITE HEMOCUE RESULTS ON HANDOUT AND GIVE TO PARTICIPANT

If participant is *not anemic*, **READ ALOUD**: The test shows that you are not anemic, thank you for your time.

If participant is *anemic*, **READ ALOUD**: The test shows that you have anemia. We would like to draw a sample of blood to find out the cause of anemia.

BLOOD DRAW (PERFORMED ONLY ON THOSE WHO ARE ANEMIC):

Completed □ Person who performed test: _______________________

After blood draw is performed, **READ ALOUD**: We have a nutritionist who will meet with you to explain how you can add food and vitamins to your diet to help you to not be anemic. Thank you for your time.
