# Supplementary material for: Individual and community factors contributing to anemia among women in rural Baja California, Mexico
Source: PLoS One. 2017 Nov 27;12(11):e0188590. doi: 10.1371/journal.pone.0188590 (PMC5703514; doi:10.1371/journal.pone.0188590)
Supplement: S2 File — This is the Spanish version of the survey administered to women in the community. (DOCX) [file pone.0188590.s002.docx]

**Prevalencia de Anemia en una colonia de migrantes en Baja California, México.**

| Nombre del entrevistador_____________________  Institución: UABC □_1_ SDSU □_2_ UCSD □_3_ | Fecha: ______________________  Lugar: Clínica □_1_ Hogar □_2_ |
| --- | --- |

***LEA EN VOZ ALTA*:** ¿Podemos hablar con la mujer adulta (15-49 años)?

**NUEVO O REEXAMINAR**

En los últimos **2 días**, ¿le han realizado exámenes de sangre (picándoles el dedo) específicamente para buscar anemia, además de haber contestado preguntas de un encuesta a alguien de esta casa en la clínica que está localizada en la escuela comunitaria o en su casa?

⬜_0_ Nuevo - La respuesta es “no”, continua con la entrevista

⬜_1_ Reexaminar- La respuesta es “sí”, **PARA** la entrevista

**LEE LA FORMA DEL CONSENTIMIENTO AL PARTICIPANTE. CONSERVE LA FORMA FIRMADA Y ENTREGUA UNA COPIA EN BLANCO PARA QUE LA CONSERVEN**

**EjERCICIO aleatorio**

|  | 1. ¿Cuantas mujeres de 15 a 49 años viven en su casa? (incluyendose a usted mismo) |
| --- | --- |
| # Viviendo en la casa |  |

si solamente hay un participante elegible, siga A **SECCION A**.

--O--

SI HAY mas de un participante elegible, **LEE en voce alta**: solamente *una persona pude estar incluido en este estudio. todos las mujeres que estan elegibles tiraran un dado y LA PERSONA QUE tira el numero mas alto estarA incluido en el estudio.*

**SECcIOn A: DATOS DEMOGRAFICOS y INFORMACION SOBRE EL HOGAR**

| 2. Iniciales de las personas viviendo en el hogar y que esta participando en la encuesta (obtenidos en el ejercicio aleatorio) | 3. Mujer  (circule) | | 4. Fecha de nacimiento  mes/día/ano | 5. Edad (escriba) |
| --- | --- | --- | --- | --- |
|  | si | no | __ __ / __ __ / __ __ | años |

favor de REGISTRARS solo UNA RESPUESTA PARA CADA PREGUNTA A MENOS QUE SE ESPECIFIQUE ALGO DIFERENTE. CIRCULE LA OPCIÓN mas APROPIADA A MENOS QUE SE ESPECIFIQUE ALGO DIFERENTE.

***LEA EN VOZ ALTA:*** *Le voy a hacer una serie de preguntas sobre su vida y de dónde usted vive.*

| 6. ¿Actualmente en que trabaja usted? (Si aplica más de una categoría seleccione lo que hace con más frecuencia.) | Trabajador del campo agrícola  Ama de casa  Educador (Ej., profesor)  Empleado en un negocios  Dueño de un negocio  Estudiante  Ninguno  Otro: Especifiqué: _________________  SE NEGó | | 1  2  6  10  11  12  13  14  99 | 6.  ______ |
| --- | --- | --- | --- | --- |
| 7.¿Cuál es su estado civil? | Soltero/ nunca casado (salta a pregunta 9)  Casado/ley común  Divorciado  Separado  Viudo  SE NEGó | 0  1  2  3  4  99 | | 7.  ______ |
| 8. ¿Cuantas personas, incluyendose a usted mismo, actualmente viven en su hogar? | (En la caja que está a la derecha, escriba el número de personas) |  | | 8.  ________ |
| 9.¿Cuantas personas viviendo en este hogar trabajan afuera de la casa? | (En la caja que está a la derecha, escribe el número de personas) |  | | 9.  _______ |
| 10. ¿Cuáles de los siguientes idiomas hablas aunque no los hable con fluidez? (Lea la lista en voz alta, seleccione todos que apliquen.)  SI: 1 NO: 0 | (a) Español  (b) Inglés  (c) Triqui  (d) Mixteca  (e)Otro: Especifiqué: _________________  SE NEGó | 1 / 0  1 / 0  1 / 0  1 / 0  1 / 0  99 | | 10.(a) ______  (b) ______  (c) ______  (d) ______  (e) ______  SI: 1 NO: 0 |
| 11. ¿Cual es el idioma que mas comúnmente se habla en su hogar? | Español  Inglés  Triqui  Mixteca  Otro: Especifiqué: _________________  SE NEGó | 1  2  3  4  5  99 | | 11.  ___________ |
| 12. ¿Puede usted leer una carta o periódico? | No  Si  SE NEGó | 0  1  99 | | 12.  ______ |
| 13. ¿Cuál es el nivel más alto de educación que usted ha completado? | Ninguno  Primaria  Secundaria  Preparatoria  Más que preparatoria  SE NEGó | 0  1  2  3  4  99 | | 13.  ________ |
| 14. ¿Cuántos cuartos hay en su casa? | # DE Cuartos |  | | 14. _______ |
| 15. ¿Usted o alguien en su casa es dueño de un coche o camión? | Si  No  SE NEGó | 1  0  99 | | 15.  ________ |
| 16. En su hogar tienen algunos de los siguientes: (Lea todas las opciones y marca las cosas con el numero apropiado) LO TENGO =1 & NO LO TENGO = 0 | (a) Electricidad  (b) Radio  (c) Televisión  (d) Refrigerador  (e) Teléfono  SE NEGó | 1 / 0  1 / 0  1 / 0  1 / 0  1 / 0  99 | | 16. (a) ______  (b) ______  (c) ______  (d) ______  (e) ______ |
| 17. ¿Qué tipo de material utiliza para cocinar en su hogar?  1=Utiliza 0=No utiliza | (a) Metal  (b) Cerámico  (c) Cerámico barnizada  Otra: Especifique: _________________  NO SE  SE NEGó | 1 / 0  1 / 0  1 / 0  1 / 0  88  99 | | 17. (a) ______  (b) ______  (c)_______  (d)_______ |

***LEA EN VOZ ALTA:*** *Ahora preguntaré sobre varios aspectos de su salud.*

**SECCion B: SALUD**

| 18. ¿Usted recibe dinero del gobierno para su alimentación y la salud del programa *Prospera*? | No  Si | 0  1 | 18.  _________ |
| --- | --- | --- | --- |
| 19. Ofrecemos una Clínica médica gratuita en la escuela, en abril y octubre cada año. ¿Alguna vez a visitado la clínica médica temporal? | No (pase a la pregunta 21)  Si | 0  1 | 19.  __________ |
| 20. ¿Cuántas veces ha visitado la clínica médica temporal en la escuela en los últimos 5 años? | (En la caja que está a la derecha, escribe el número) |  | 20.  __________ |
| 21. ¿Alguna vez ha cambiado su hoyas de barnizada cerámico por utensilios nuevos de cocina durante el tiempo que han vivido en esta comunidad? | No  Si | 0  1 | 21.  ­­­  __________ |
| 22. ¿Alguna vez ha recibido educación sobre nutrición o recibido recetas saludables durante el tiempo que han vivido en esta comunidad? | No  Si | 0  1 | 22.  ­­­  __________ |

| 23. ¿Cómo calificaría usted su estado de salud? (Lea todas las opciones y seleccione una de las siguientes) :  0=Malo 1=Regular 2=Bueno 3=Excelente | 23.  ______ |
| --- | --- |
| 24. ¿En los últimos TRES MESES, usted tiene algunos de los siguientes síntomas? *(*CIRCULA ‘SI’ O ‘NO’) | S=si N=no |
| a. Pérdida de peso sin explicación | S _1_ n _0_ |
| b. Debilidad | S _1_ n _0_ |
| c. Fatiga | S _1_ n _0_ |
| d. Falta de aire | S _1_ n _0_ |
| e. Dolor de cabeza | S _1_ n _0_ |
| f. Mareo | S _1_ n _0_ |
| g. Irritabilidad | S _1_ n _0_ |
| h. Piel pálida | S _1_ n _0_ |
| i. Ritmo cardíaco aumentado | S _1_ n _0_ |
| j. Diarrea. (3 o más veces o excrementos sueltos, acuosos o ensangrentadas cada día.) | S _1_ n _0_ |

**SECCION C: SALUD REPRODUCTIVA**

| 25. ¿En este momento está usted embarazada? | Si  No  No se | 1  0  88 | 25.  ______ |
| --- | --- | --- | --- |
| 26. ¿Usted menstrua actualmente? (no esta en la menopausia o antes de la menarquia) | Si  No | 1  0 | 26.  ______ |
| 27. ¿Cuántas veces en tu vida has estado embarazada, si tuvo o no tuvo el bebé? | REGISTRAR # DE EMBARAZOS  (Si nunca, ponga '0 '& pase a SECTION D) |  | 27.  ______ |
| 28. ¿Ha dado a luz en los últimos seis meses? | Si  No | 1  0 | 28.  ______ |
| 29. ¿Actualmente esta amamantando (dando pecho)? | Si  No  NO APPLICA | 1  0  77 | 29.  ______ |

**SECCION D: comida**

| 30. | *Para las siguientes preguntas, favor de decirme si usted comió cada comida en los últimos 48 horas.* | **Si_1_** | **No_0_** |
| --- | --- | --- | --- |
| a. | Pescado o mariscos | □_1_ | □_0_ |
| b. | Carne | □_1_ | □_0_ |
| c. | Pavo, pollo, puerco | □_1_ | □_0_ |
| d. | Huevo | □_1_ | □_0_ |
| e. | Cereal seco o avena instantánea | □_1_ | □_0_ |
| f. | Nueces o semillas | □_1_ | □_0_ |
| g. | Frijoles, lentejas, y legumbres | □_1_ | □_0_ |
| h. | Fruta seca | □_1_ | □_0_ |
| j. | Arroz, pan, pasta, tortillas | □_1_ | □_0_ |
| k. | Vegetales Verde (Espárragos, brócoli, chícharos, espinaca) | □_1_ | □_0_ |
| l. | Té | □_1_ | □_0_ |
| m. | Café | □_1_ | □_0_ |
| n. | Jugo de naranja o cítricos (naranjas, limas, toronjas) | □_1_ | □_0_ |
| o. | Alcohol (cerveza, vino, licores fuertes) | □_1_ | □_0_ |
| p. | Vitaminas | □_1_ | □_0_ |

***LEA EN VOZ ALTA:*** *Gracias, ahora nos gustará hacerle a usted una prueba de sangre para determinar si usted es anémico.*

****SECTION E: hEMOCUE RESULTS****

| 31. Resultados de la prueba de Hemocue (escriba el número de la hemocue) |  |
| --- | --- |
| 32. ¿Fue referido el individuo para una prueba de sangre por ser anémico? | s_1_ n _0_ |

PRUEBA DE HEMOCUE:

Número de la máquina de HemoCue: ____________ Persona que hizo el examen: _____________

ESCRIBE RESULTADOS DEL HEMOCUE EN EL FOLLETO Y ENTREGALO AL PARTICIPANTE

Si el participante *no es* *anémico*, **LEA EN VOZ ALTA:** Esta prueba demuestra que usted no es anémico, gracias por su tiempo.

Si el participante *es* *anémico*, **LEA EN VOZ ALTA:** Esta prueba demuestra que usted es anémico. Nos gustará hacerle una prueba de sangre para averiguar la causa de la anemia.

PRUEBA DE SANGRE (SOLO PARA LOS PARTICIPANTES QUE SON ANÉMICOS):

Persona que hizo el examen: ____________________

Después de hacer la prueba de sangre, **LEA EN VOZ ALTA:** En este momento tenemos aquí un nutricionista que va hablar contigo para explicar cómo usted puede añadir comidas y vitaminas a su dieta para ayudarle no ser anémico. Gracias por su tiempo.
